# Efficacy of non-psychotropic *Cannabis sativa* L. standardized extracts in a model of intestinal inflammation

**DOI:** 10.1186/s42238-025-00335-2

**Published:** 2025-10-06

**Authors:** Nicole Maranta, Giulia Martinelli, Marco Fumagalli, Carola Pozzoli, Elisa Sonzogni, Nora Rossini, Umberto Ciriello, Giuseppe Paladino, Mario Dell’Agli, Stefano Piazza, Enrico Sangiovanni

**Affiliations:** 1 Università degli Studi di Milano, Milan, Italy; 2 Linnea SA, Riazzino, Switzerland

**Keywords:** Cannabis, Gut inflammation, IBD CBD, CBG

## Abstract

**Background:**

The use of *Cannabis sativa* L. (*Cannabis*) was reported by observational studies on inflammatory bowel diseases (IBD) patients. However, this indication is poorly supported by clinical trials. Several pre-clinical studies demonstrated the anti-inflammatory activity of Δ⁹-tetrahydrocannabinol (Δ⁹-THC) and cannabidiol (CBD) at intestinal level. On the contrary, minor cannabinoids, such as cannabigerol (CBG), were less investigated. Moreover, several authors suggested that complex *Cannabis* extracts might display a higher efficacy in respect to pure cannabinoids against inflammatory disorders.

**Methods:**

This study was aimed at investigating the role of *Cannabis* extracts, standardized in CBD and CBG content, in a model of in vitro-induced intestinal inflammation using CaCo-2 cells. Inflammatory mediators at transcriptional (PCR arrays) and protein level (ELISA assays) were investigated and correlated with enterocyte layer permeability. The two evaluated extracts, A and B, come from the mix of the same *Cannabis* varieties (*Cannabis sativa* L. Chemotype III and Chemotype IV), and are standardized in CBD and CBG at the same level, by changing the polarity of the primary extraction solvents.

**Results:**

Pro-inflammatory cytokines involved in IBD, such as IL-1β and IFN-γ, induced the expression and the release of chemokines for lymphocytes (CXCL-9, CXCL-10, CCL20) in CaCo-2, while *Cannabis* extracts (100 µg/mL) or individual compounds (8 µM) showed inhibitory activity. After simulated digestion, extract A abrogated the release of CCL-20, while extract B abrogated the release of CXCL-9 and CXCL-10. The inhibition of CXCL-9 was demonstrated at transcriptional level also. The inhibitory activity paralleled with the content of CBD or CBG, acting at least in part through NF-κB impairment (-42% and − 66%, respectively). However, *Cannabis* extracts showed greater effect in the CaCo-2-THP-1 co-culture inflammation model compared to individual cannabinoids, thus partially recovering the epithelial barrier measured by transepithelial electrical resistance (TEER), and zonula occludens (ZO-1) expression.

**Conclusions:**

Data collected within this study showed the importance of standardization and extraction method reproducibility through manufacturing and process control, besides demanding future investigations focusing on the effect of *Cannabis* extracts against intestinal inflammation, which show in this context effects higher than individual cannabinoids.

**Supplementary Information:**

The online version contains supplementary material available at 10.1186/s42238-025-00335-2.

## Background

Non-psychotropic compounds from *Cannabis sativa* L. (*Cannabis*) are reaching a growing attention for their pharmacological properties and safety profile (Turner et al. 2017; Friedman et al. 2019). They interact with the endocannabinoid system and other receptors involved in inflammation and pain, such as TRPs and PPARs (Bisogno et al. 2001; Friedman et al. 2019). Among the most abundant non-psychotropic compounds, cannabidiol (CBD) was extensively investigated and found clinical application in neurological disorders and pain (Giacoppo, Bramanti, and Mazzon 2017; Georgieva et al. 2023).

One of the critical aspects related to the use of cannabinoids for medicinal purpose is their pharmaceutical quality, since the interaction among cannabinoids may influence the biological activity of the final product (Koturbash and MacKay 2020). Beside CBD, other bioactive cannabinoids, such as cannabigerol (CBG), cannabichromene (CBC), cannabinol (CBN), cannabidivarin (CBDV), and natural compounds, such as terpenes (e.g. β-caryophillene), and flavonoids (e.g., cannflavines) were reported in *Cannabis* extracts (Caprioglio et al. 2022; Erridge et al. 2020).

*Cannabis* and its derivatives are increasingly demanded and consumed by people suffering from abdominal pain due to inflammatory bowel disease (IBD); this indication is inferred from empirical evidence of life quality improvement, but it is still poorly sustained by clinical trials (Neufeld et al. 2021; Velez-Santiago et al. 2023). As mentioned, the quality of *Cannabis*-based products used in IBD patients, their origin and posology are nonuniform (Velez-Santiago et al. 2023; Hasenoehrl, Storr, and Schicho 2017). Moreover, their consumption is under-reported to physicians and might regard, at least in part, products from non-pharmaceutical markets, including the black market (Neufeld et al. 2021), thus raising concerns about quality and, consequently, safety and efficacy. For example, it is plausible that non-monitored products might contain undefined amounts of psychoactive molecules, such as Δ⁹-tetrahydrocannabinol (Δ⁹-THC), which is an obvious matter of concern, beyond the risk to invalidate therapy success.

Nevertheless, examples of promising clinical studies, conducted with standardized *Cannabis* extracts, are available from the literature. These studies focused on the role of Δ⁹-THC and CBD for the control of pain, inflammation, and anxiety disorders related to IBD (Naftali et al. 2013, 2021; Irving et al. 2018). The anti-inflammatory properties of Δ⁹-THC and CBD at intestinal level were demonstrated by experiments in epithelial cells or rodent models of colitis (Pagano et al. 2016; Sun et al. 2024; Cocetta et al. 2021; Schicho and Storr 2012; Jamontt et al. 2010). On the contrary, the role of CBG as further anti-inflammatory and non-psychotropic compound common in *Cannabis* extracts was less investigated (Borrelli et al. 2013; Anderson et al. 2024; Marsh and Smid 2024), although showing similarities for the activity profile when compared to Δ⁹-THC.

It is noteworthy that the combination of Δ⁹-THC and CBD has been reported to enhance the bioactivity in respect to separate compounds (Jamontt et al. 2010). Similarly, several authors, including our group (Sangiovanni et al. 2019; Martinelli et al. 2022), suggested that complex *Cannabis* extracts might display efficacy higher than individual cannabinoids against inflammatory disorders (Anil, Peeri, and Koltai 2022; Yekhtin et al. 2022).

Based on these premises, the present study aimed at investigating the anti-inflammatory properties of two standardized *Cannabis* extracts, with low THC (below 0.2%), at intestinal level. For the purpose, experiments were carried out in different models of intestinal inflammation, involving human colonocytes (undifferentiated CaCo-2 cells) and enterocytes (differentiated CaCo-2 cells) challenged with pro-inflammatory cytokines typical of IBD disease. The combination of IL-1β and IFN-γ was used to reproduce innate immunity and type 1 acquired immunity (Van De Walle et al. 2010), respectively, thus stimulating inflammatory pathways involved in autoimmune disorders, such as NF-κB and STAT (Guan 2019). In models of intestinal inflammation, IFN-γ is also required to cause the increase of epithelial permeability and the alteration of tight junctions (Huang et al. 2021). The bioactivity of *Cannabis* extracts was compared within the same context with that of the major components CBD and CBG.

## Materials and methods

### Materials

Cannabidiol (CBD), Cannabigerol (CBG), and *Cannabis* extracts, were kindly provided by Linnea SA (Riazzino, Switzerland). Lipopolysaccharide from *E. coli* O111:B4 (LPS), phorbol-myristate acetate (PMA), sodium butyrate, high glucose DMEM, 3,4,5-dimethylthiazol-2-yl-2-5-diphenyl-tetrazolium bromide (MTT), inorganic salts or digestive enzymes for in vitro simulated digestion, were purchased from Sigma-Aldrich (Merck Life Science, Milano, Italy). RMPI medium, Trypsin-EDTA 0.25%, streptomycin, penicillin, non-essential amino acids, sodium pyruvate, and L-glutamine were from Gibco™ (Thermo Fisher Scientific, MA, USA), while cell plates and other disposable materials (Primo^®^ or Falcon^®^), and fetal bovine serum (FBS) were from Euroclone (Euroclone S.p.a., Pero, Italy). Bioactive molecules and extracts used for experiments were dissolved in DMSO and stored at -20 °C until cell treatments.

Human pro-inflammatory cytokines (IL-1β and IFN-γ) and human ELISA assay kits for CXCL-9 and CXCL-10 were from Peprotech (Thermo Fisher Scientific, Waltham, MA, USA), while CCL-20 and IL-15 assay kits were from BioGems (BioGems, Westlake Village, CA, USA) and Raybiotech (Raybiotech, Peachtree Corners, GA, USA), respectively. Plasmid transfection kit was from Invitrogen^®^ (Thermo Fisher Scientific, Waltham, MA, USA). The plate reader used for the acquisition of absorbance or luminescence (VICTOR X3), and Britelite™ Plus reagent (luciferin) were from Perkin Elmer (Perkin Elmer, Milano, Italy).

Methanol, and HPLC-grade water were from CARLO ERBA Reagents, Cornaredo, Italy.

### Cannabis extracts preparation

All the cannabinoids and the extracts are manufactured at Linnea SA facility and come from *Cannabis* selected and stabilized strains. Both the A and B extracts are standardized, with CBD and CBG in 1:1 proportion and 5% w/w.

The CBD and CBG concentrations have been measured through internal and validated HPLC/UV analytical method. THC levels are as well quantified as lower than 0.2% for both the extracts. Moreover, terpenes were identified and quantified through an internal and validated GC/FID analytical method (Tab. S1).

To test the impact of the extraction method on the stability through the digestion and the consequent biological activity, CBG-rich and CBD-rich *Cannabis* aerial parts have been extracted using solvents with different polarity: extract A is a blend of two *Cannabis* extracts treated with absolute ethanol, while extract B is obtained using a mix of ethanol and water 50%.

After extraction, the decarboxylation at high temperature occurred, then extracts are filtrated, and residual solvents eliminated (solvent elimination is verified through GC analysis). The extracts are finally blended and standardized with MCT oil, Ph. Eur. grade, at 5%.

### LC-MS analysis of cannabis extract

For the purpose of this experiment, Cannabidiol (CBD) and Cannabigerol (CBG) were quantified using an LC–MS/MS system. HPLC was performed through an Exion LC^™^ AC System (AB Sciex, Foster City, CA, USA) composed of a vacuum degasser, a double plunger pump, a cooled autosampler, and a temperature-controlled column oven. The MS/MS analysis was carried out with a Triple Quad^™^ 3500 system (AB Sciex, Foster City, CA, USA). The analytes were separated using a Synergi 4 μm Hydro-RP 80 Å LC Colum 150 × 4.6 mm column (Phenomenex, Torrance, California, USA) with a mobile phase composed of 0.1% formic acid in water (A) and methanol (B) at a rate flow of 0.800 mL/min. The chromatographic gradient was set as described in Table 1.

**Table 1:** Chromatographic gradient used for analysis by LC–MS

Time (min)	% (A)	% (B)
0	40	60
1	40	60
10	0	100
12	0	100
12.1	40	60
17	40	60

The injection volume was 10 µL for each sample. Mass spectrometric detection was done in negative ionization (ESI) mode, and the parameters were set as follows: curtain gas at 40 psi, ionization voltage at − 4500 V, source temperature at 500 °C, and nebulization gas 1 and nebulization gas 2 at 50 psi.

The optimized compound-dependent MS/MS parameters (declustering potential, entrance potential, collision energy, and collision cell exit potential) were obtained in multiple-reaction-monitoring (MRM) mode by a separate infusion of the analytes. The analytes were quantified by using the following mass transitions: 313/245 (cannabidiol), 315/136 (cannabigerol). The LC–MS/MS system was controlled by AB Sciex Analyst (version 1.7) software.

### Cell culture and treatments

Cells from human colorectal adenocarcinoma (Caco-2, clone HB237) or human monocytic leukemia (THP-1) were purchased from ATCC (ATCC, VA, USA). CaCo-2 cells were cultured with high glucose DMEM containing 100 mg streptomycin, 100 units penicillin, 1% non-essential amino acids, 1 mM sodium pyruvate, 4 mM L-glutamine and 10% FBS. THP-1 cells were cultured in suspension with RPMI medium containing 100 mg streptomycin, 100 units penicillin, 4 mM L-glutamine, and 10% FBS.

Cells were sub-cultured every 48–72 h in 75 cm^2^ flasks, under a humidified atmosphere with 5% CO_2_ at 37 °C. Adherent cells (CaCo-2) were detached using Trypsin-EDTA 0.25% before centrifugation and manual counting with Bürker chamber.

To obtain enterocytes-like cells, CaCo-2 cells were cultivated on Transwell^®^ support (12 wells, 3 × 10^5^ cells/well) until day 17 to 21. During the differentiation time, complete DMEM medium was added to the basolateral compartment, while FBS-free medium was added to the apical compartment, every other day. To obtain the respective co-culture with human macrophages, THP-1 cells (2 × 10^5^ cells/well) were previously differentiated in 12-well plates by phorbol-ester (PMA, 25 µM) for 48 h, thus obtaining adherent macrophages; then, Transwell^®^ supports containing enterocytes were transferred onto macrophages.

All treatments were carried out using DMEM FBS-free medium, in which pro-inflammatory cytokines were concomitantly added to extracts or cannabinoids under study. More in detail, CaCo-2 cells were grown in 24-well plates (3 × 10^4^ cells/well) for 48 h and stimulated for the specified periods of time (6–24 h) by IL-1β/IFN-γ (10 ng/mL, each); conversely, the co-culture was stimulated by LPS (100 ng/mL)/IFN-γ (10 ng/mL) (at the basolateral side) and treated with extracts or pure cannabinoids for 48 h (at the apical side) (Sonzogni et al. 2024; Pozzoli et al. 2024).

The concentrations of extracts (100 µg/mL) and compounds (8 µM) were selected by our previous studies, suggesting the potential bioactivity at epithelial level (Fumagalli et al. 2025; Sangiovanni et al. 2019). Moreover, these concentrations were considered as theoretically achievable at intestinal level after oral ingestion.

### MTT assay

Cell morphology was observed at the end of each experiment by light microscope inspection. Cell viability was measured by MTT assay at the end of cell treatment, as previously described (Sonzogni et al. 2024). In brief, MTT reagent was diluted into PBS 1X solution reaching the final concentration of 200 µg/mL. The solution was added to cells after removing culture media; then, after 15–30 min of incubation, a lysis buffer (isopropanol: DMSO, 90:10) was used to dissolve the intracellular purple salt of formazan, thus obtaining samples with absorbance at 550 nm. The absorbance of tested samples was compared to the stimulated control: a relative absorbance lower than 80% was arbitrarily considered a potential index of cytotoxicity.

### ELISA assay

Human ELISA assays were performed following manufacturer’s instructions, as reported in previous articles (Sonzogni et al. 2024). The pre-coated plates were used directly to measure the presence of IL-15 or CCL20 in cell media collected after 24 h of treatment (100 µL), while the CXCL-9 and CXCL-10 assay kits required a preliminary passage with the coating antibody incubated overnight. Then, biotinylated antibodies targeted to each inflammatory protein were added for 1–2 h, according to specifications. Finally, the avidin-HRP construct was added to mediate the oxidation of the ABTS substate into a green product, measured by plate reader at absorbance of 405 nm. The amount of inflammatory mediator (pg/mL) was calculated by comparison with a calibration curve of each standard protein (ranging from 0 to 2000 pg/mL). Data were expressed as relative percentage (%) to stimulated control, which was arbitrarily assigned to the value of 100%.

### Luciferase assay

The day before treatment, CaCo-2 cells were transiently transfected with a plasmid containing NF-κB responsive elements (100 ng per well), by following Invitrogen^®^ Lipofectamine 3000 Reagent protocol (Sonzogni et al. 2024). The plasmid (NF-κB-Luc) was a kind gift from Dr. N. Marx (Department of Internal Medicine-Cardiology, University of Ulm; Ulm, Germany). The activity of the inflammatory transcription factors was evaluated after 6 h of treatment with cannabinoids (8 µM) or *Cannabis* extracts (100 µg/mL) under study and pro-inflammatory cytokines. At the end of treatments, Britelite™ Plus reagent, bringing luciferin and lysis agents, was added to cell layer, thus developing luminescence. Light units measured by plate reader were directly correlated with the activation of NF-κB; thus, data were expressed as % of NF-κB driven transcription with respect to stimulated control (100%).

### RNA extraction and PCR array

RNA was isolated from CaCo-2 cells through NucleoSpin^®^ RNA Plus extraction kit (Macherey-Nagel, Düren, Germany), according to manufacturer’s instructions. In brief, cells were homogenized and lysed by a ready-to-use sample buffer (350 µL) after 24 h of treatment; then, DNA was removed by exclusion columns centrifuged at 11’000 g for 30 s. The RNA contained in the flowthrough was bound onto another silica column, while other biological interferences were discarded through centrifugation (11’000 g for 15 s). RNA samples were washed three times on column by using different washing buffers and centrifugation passages; finally, silica membranes were dried by prolonged centrifugation (11’000 g for 2 min) to proceed with RNA elution into RNAase free water (60 µL). The amount of RNA and its quality were measured by NanoDrop ND-1000 (Thermo Fisher Scientific, Waltham, MA, USA).

The transcription of inflammatory genes was measured by PCR RT² Profiler™ (“PCR Array Human Inflammatory Cytokines & Receptors”, QIAGEN, Hilden, Germany), including 84 target and 5 housekeeping genes. Moreover, 3 reverse transcription control, and 3 positive PCR control were included. In brief, cDNA was synthetized by RT^2^ First Strand kit, starting by 400 ng of RNA sample, and used for PCR analysis, according to the manufacturer’s instructions (QIAGEN). The obtained cDNA was mixed with the SYBR Green Master Mix RT^2^ reagent and loaded into the 384-well profiler. The amplification and quantification was performed by C1000™ Thermal Cycler coupled with CFX384™ Real‐Time PCR Detection System (Bio‐Rad Laboratories, Segrate, Italy), as previously described (Sonzogni et al. 2024).

C^T^ values were exported to an Excel template to create a table of C^T^ values, with cut-off set to 35, following manufacturer’s web portal SABiosciences (QIAGEN). This table was then uploaded onto the data analysis web portal at http://www.qiagen.com/geneglobe. Samples were assigned to controls and tests groups. C^T^ values were normalized based on a manual selection of the most reproducible housekeeping genes. The web platform calculated fold change using delta delta C^T^ method, in which delta C^T^ is calculated between inflammatory genes of interest and an average of housekeeping genes, followed by delta − delta C^T^ calculations (delta C^T^ of Test Group − delta C^T^ of Control Group). Fold Change is then calculated using 2^(− delta delta C^T^) formula. The data analysis web portal also generated scatter plots and tables reporting the Fold Changes and the p values.

### Measurement of the epithelial integrity

The integrity of enterocyte monolayer (CaCo-2 grown on Transwell^®^ support) was indirectly measured by trans-epithelial electrical resistance (TEER, Ω), using EVOM3 device (WPI, Sarasota, FL, USA), as previously described (Sonzogni et al. 2024). The reduction of TEER values was interpreted as impaired barrier integrity. Measures were taken before and after each treatment and repeated three times, with a threshold value at the beginning set above 400 Ω. *Cannabis* extracts and molecules (CBD and CBG) were added simultaneously to the pro-inflammatory stimuli in the apical compartment (respectively 100 µg/mL, and 8 µM). Sodium butyrate (2 mM) was used as a well-known reference inhibitor of barrier impairment (Huang et al. 2021). Eventually, we calculated the variation of the TEER (ΔΩ) by comparing values before and after treatments, as follows: ΔΩ = Ωt_24h_ − Ωt_0_. Results were expressed as relative data to the stimulated condition, which was assigned the value of 0.

### Immunofluorescence

ZO-1 (zonulin-1) expression was evaluated on CaCo-2/THP-1 co-culture, after 48 h of treatment with *Cannabis* extracts and pure molecules (CBD and CBG), in addition to the pro-inflammatory stimulation. Sample were obtained by fixation with formaldehyde, as previously reported (Sonzogni et al. 2024). In brief, at the end of the treatment the medium was discarded, and enterocyte monolayer was mounted on glass slides for the immunostaining. After a first wash with 500 µL of PBS 1X, the cells were allowed to fix for 15 min with 500 µL of PBS 1X/PFA 4%; then, after three wash of 5 min each (PBS 1X), the membranes were cut from the Transwell^®^ support and moved into a new well, where 500 µL of blocking buffer (1X PBS with 5% BSA, 0.3% Triton X-100) were added for 1 h. At the end of the incubation period, the buffer was discarded, and 400 µL of the primary rabbit anti-human ZO-1 antibody (1 mg/mL, cat. #13663, Cell signaling, Danvers, MA, USA), with dilution of 1:400, was added and incubated overnight at 4 °C. The following day, after three new rinses of 5 min each with PBS 1X, the membranes were incubated with 400 µL of the fluorochrome AlexaFluor^®^ 647-conjugated anti-rabbit antibody (1 mg/mL, cat. #8889S, Cell signaling, Danvers, MA, USA), with a dilution of 1:800, for 2 h. Eventually, after other three rinse with PBS 1X, they were mounted on the glass slides using the ProLong Gold Antifade DAPI (Cell Signaling, Danvers, MA, USA), used as a fixative and nuclei stainer. Samples were covered with the coverslip and images were finally acquired by confocal microscopy (LSM 900, Zeiss, Oberkochen, Germany), with a 60X magnification.

### Statistical analysis

All biological data were normalized and expressed as mean ± SEM; at least three experiments (*n* = 3) in which measures were acquired twice for each sample were conducted. Data were checked for normality and homogeneity of variances. Then, data with normal distribution without equal variance were analyzed by Brown-Forsythe and Welch ANOVA followed by Dunnett T3 multiple comparison test, while data without normal distribution were analyzed by nonparametric ANOVA (Kruskal-Wallis test) followed by Dunn’s multiple comparison test. Calculations were performed by GraphPad Prism 9.0 software (GraphPad Software, San Diego, CA, USA), setting *p* < 0.05 as statistically significant. Calculations concerning data from PCR array were analyzed by data analysis web portal at http://www.qiagen.com/geneglobe, as previously described.

## Results

Cannabis extracts A and B were standardized by Linnea SA, with CBD and CBG in 1:1 proportion and 5% w/w. Moreover, GC/FID analysis was provided, indicating terpenes as a minor fraction: total terpenes were 0.61% and 0.19% of Cannabis extract A and B, respectively (Tab. S1). The first step of the experimental work was an additional quantification of CBD and CBG in both *Cannabis* extracts (A and B) with low THC, by LC-MS analysis. As expected from the batch release analysis, the two extracts differing from the extraction methods, showed instead comparable amounts of CBD, ranging from 3.747% to 4.167%, and CBG, ranging from 3.103% to 3.667%, respectively (Table 2). Moreover, the amount of cannabinoids was only slightly altered after a simulation of the gastrointestinal digestion, with a relatively major impact on extract A in respect to extract B.

**Table 2:** LC-MS/MS quantitative analysis of the content of CBD and CBG in extract A and extract B subjected to simulated Gastrointestinal digestion

Cannabis sativa L. extract	CBD (% w/w) ± SEM	CBG (% w/w) ± SEM
Extract A	3.747 ± 0.632	3.103 ± 0.018
Extract A (Dig.)	2.637 ± 0.319 ^N.S^.	2.013 ± 0.127 ^N.S^.
Extract B	4.167 ± 0.575	3.667 ± 0.081
Extract B (Dig.)	3.737 ± 0.774 ^N.S^.	3.453 ± 0.805 ^N.S^.

Dig., gastrointestinal simulated digestion; N.S., not significantly different from undigested extract

Subsequently, the bioactivity of *Cannabis* extracts was investigated in human colonocytes (CaCo-2 cells) stimulated by IL-1β and IFN-γ, selected as pro-inflammatory cytokines involved in IBD (Guan 2019). CBD and CBG were tested at a concentration very close to that present in *Cannabis* extracts, for comparison.

Concurrently, potential cytotoxicity was measured by MTT assay, which excluded alterations in cell viability up to the concentration of 100 µg/mL of extracts (Fig. S1a). On the contrary, the treatment with pure CBD or CBG led to a slight reduction of cell viability (-25% and − 18%, respectively) at the concentration of 8 µM, while the sum of the two cannabinoids (1:1) at the same concentration strongly reduced it (-7 9%). Consequently, the anti-inflammatory activity of *Cannabis* extracts was investigated by selecting the concentration of 100 µg/mL, in comparison with 8 µM pure cannabinoids (corresponding to an approximate amount of 2.5% *w/w* of extract).

Following previously published experimental settings (Pozzoli et al. 2024; Sonzogni et al. 2024), inflammatory mediators playing central role in lymphocytes recruitment, such CXCL-10, and the related transcription factor NF-κB, were selected as reliable read-outs.

**Fig. 1:**
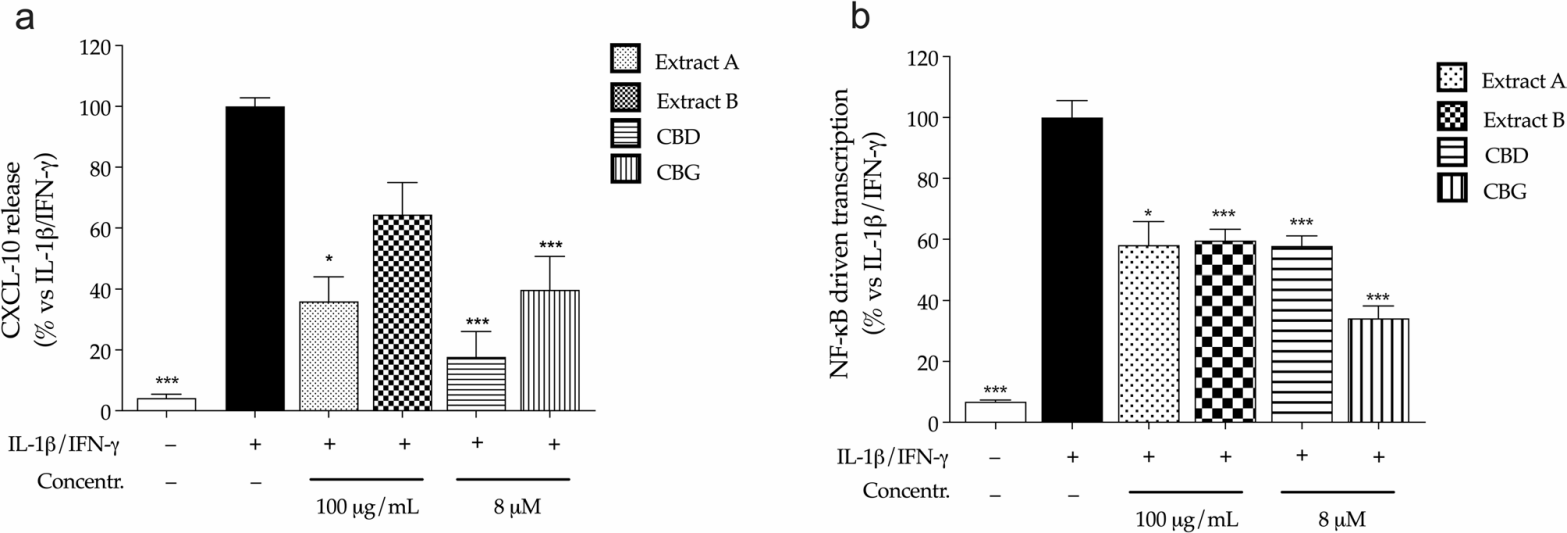
Effect of *Cannabis* extracts (Extract A, Extract B) and pure cannabinoids (CBD, CBG) on the release of CXCL-10 (**a**) and the activation of NF-κB (**b**) in colonocytes (CaCo-2). CXCL-10 release was measured by ELISA (24 h), while NF-κB driven transcription was measured by luciferase assay (6 h). Cells were treated with extracts (100 µg/mL) or pure molecules (8 µM) in addition to inflammatory stimuli (IL-1β/IFN-γ), which value was arbitrarily assigned to 100%. Sodium butyrate 2 mM was used as reference inhibitor of NF-κB activity (-55%) and CXCL-10 release (-30%). Data are expressed as average (%) ± SEM (*n* = 3). * *p* < 0.05; ***p < 0.001 (Kruskal-Wallis, and Brown-Forsythe and Welch test, respectively) vs. IL-1β/IFN-γ

Extract A and B (100 µg/mL) counteracted the release of CXCL-10 in CaCo-2 cells, although the effect was significant for extract A, only (Fig. 1a). Moreover, a significant inhibitory effect was observed in experiments concerning the transcriptional activity of NF-κB (Fig. 1b), thus suggesting its involvement in the mode of action of the extracts. The resulting degree of inhibition was comparable to that of connabinoids: the bioactivity was very similar to extract A referring to both inflammatory markers. These experiments clearly suggested the involvement of the two cannabinoids in the pharmacological activity of *Cannabis* extracts.

**Fig. 2:**
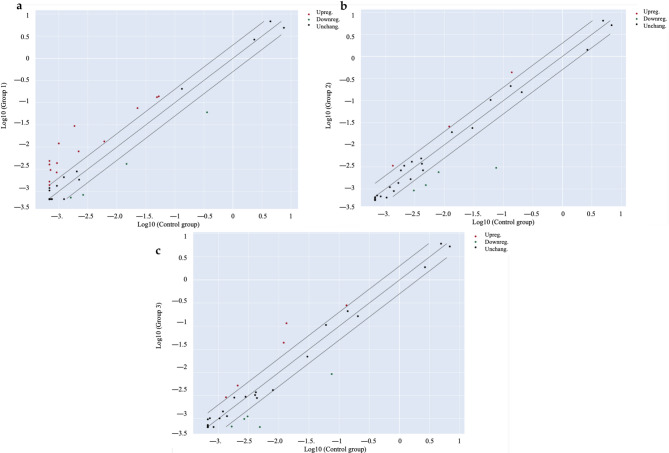
Scatter plots of the expression profile of inflammatory genes measured by PCR array in colonocytes (CaCo-2). Cells were treated for 24 h with *Cannabis* extracts (100 µg/mL) underwent to simulated digestion, in addition to inflammatory stimuli (IL-1β/IFN-γ). Plots of unstimulated control (**a**), Extract A Dig. (**b**), and Extract B Dig. (**c**) versus IL-1β/IFN-γ are reported, respectively. The center diagonal line indicates unchanged gene expression (black dots), while the outer diagonal lines indicate the selected fold regulation threshold. Genes with data points beyond the outer lines in the upper left and lower right corners are up-regulated (red dots) or down-regulated (green dots), respectively, by more than the fold regulation threshold in the y-axis Group relative to the x-axis Group. Scatter plots were automatically generated by GeneGlobe web portal analysis

**Fig. 3:**
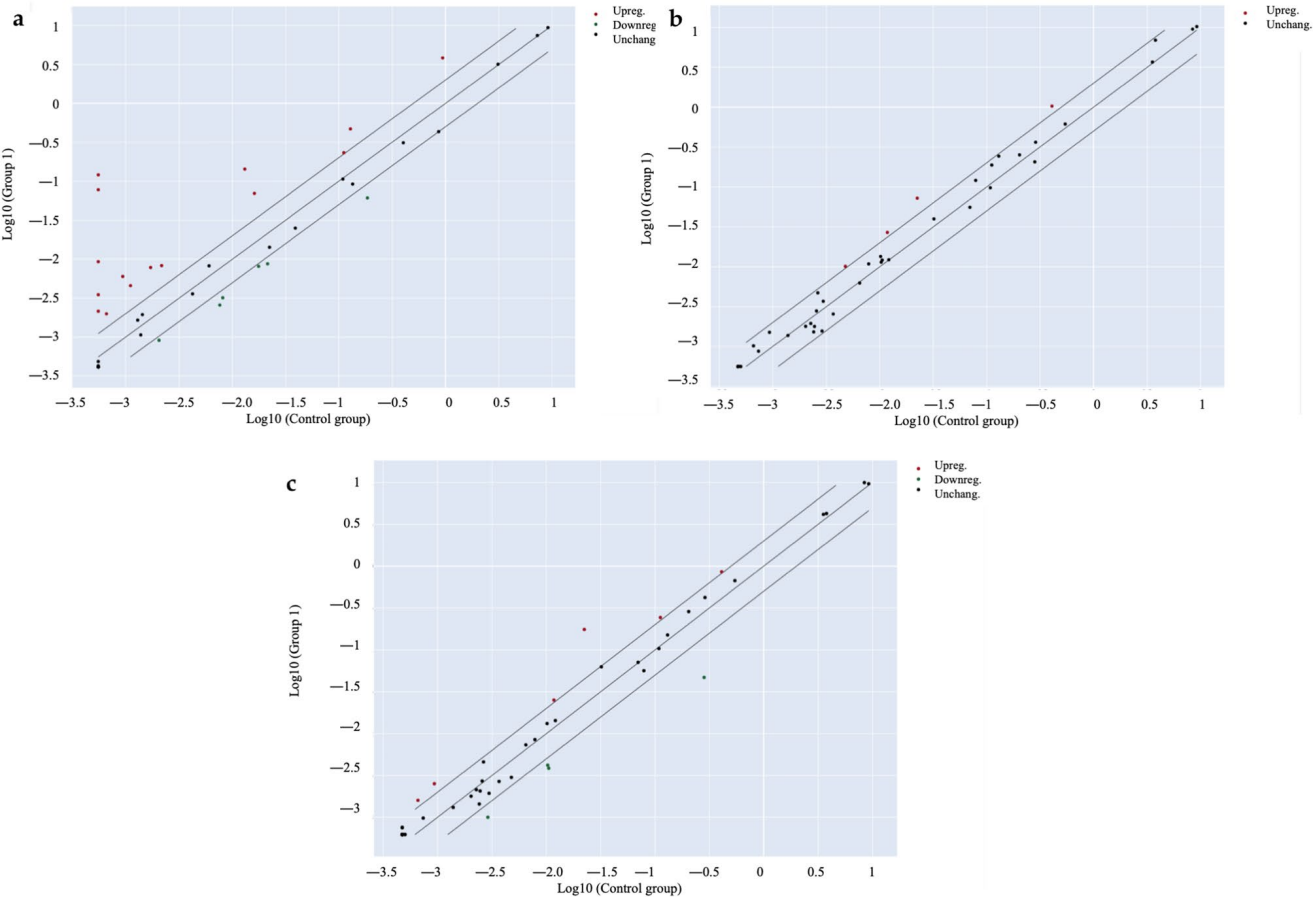
Scatter plots of the expression profile of inflammatory genes measured by PCR array in enterocytes (differentiated CaCo-2). Cells were treated for 24 h with *Cannabis* extracts (100 µg/mL) subjected to simulated digestion, in addition to inflammatory stimuli (IL-1β/IFN-γ). Plots of unstimulated control (**a**), Extract A Dig. (**b**), and Extract B Dig. (**c**) versus IL-1β/IFN-γ are reported, respectively. The center diagonal line indicates unchanged gene expression (black dots), while the outer diagonal lines indicate the selected fold regulation threshold. Genes with data points beyond the outer lines in the upper left and lower right corners are up-regulated (red dots) or down-regulated (green dots), respectively, by more than the fold regulation threshold in the y-axis Group relative to the x-axis Group. Scatter plots were automatically generated by GeneGlobe web portal analysis

As a second step, the bioactivity was more deeply investigated by considering the stability of cannabinoids in extracts underwent to simulated digestion, as underlined in Table 2. Once again, the cytotoxicity of digested extracts was excluded (Fig. S1b). Furthermore, the investigation of digested *Cannabis* extracts (Extract A Dig. and Extract B Dig.) were enhanced by using a PCR array approach. The transcription of inflammatory genes related to human cytokines and receptors was measured in either colonocytes (undifferentiated CaCo-2) and enterocytes (differentiated CaCo-2 on Transwell^®^ plates) after 24 h of stimulation with IL-1β and IFN-γ. As expected, the pro-inflammatory challenge caused the over-expression of several inflammatory genes in both models (Figs. 2a and 3a). The transcription pattern was similar and included genes belonging to the CXC- class (*CXCL2*, *CXCL3*, *CXCL8*, *CXCL9*, *CXCL10*), *CCL20* and its receptor *CCR6*, *CSF1*, and *IL15* (Table 3, Tab. S2). However, *Cannabis* extracts were able to inhibit the over-expression of several genes in colonocytes (Fig. 2b and c), while the inhibition was not relevant in enterocytes (Fig. 3b and c). Additional data were obtained by measuring further selected markers at protein level by ELISA assay. ELISA confirmed that neither extracts nor cannabinoids modulated IL-15 release or chemokine secretion in enterocytes consistent with transcriptional data (Fig. S2, Fig. S3).

On the contrary, several CXC- genes were downregulated in colonocytes, showing a significant inhibition for *CXCL9* gene (Table 3). This observation, along with previous results concerning CXCL-10 release, suggested a potential drawback in the recruitment of Th1 lymphocytes at colon level.

**Table 3:** Summary of gene expression measured by PCR array in colonocytes (CaCo-2)

Genes over-expressed vs. IL-1β/IFN-γ																			
Unstimulated control							Extract A Dig.								Extract B Dig.				
Gene Symbol	Fold Reg.		*p*-Value				Gene Symbol			Fold Reg.		*p*-Value			Gene Symbol		Fold Reg.		*p*-Value
*CCL20*	12.07		0.108507				*CCL20*			2.15		0.629056			*CCL20*		3.65		0.141192
*CCR6*	3.63		**0.043053**				*TNFRSF11B*			2.52		0.405113			*IL10RB*		2.51		0.282803
*CSF1*	4.66		**0.010860**				*B2M*			3.14		0.160604			*NAMPT*		2.12		**0.029328**
*CXCL10*	2.00		0.361713												*TNFRSF11B*		2.18		0.450456
*CXCL2*	15.96		0.057732																
*CXCL3*	4.21		0.323904																
*CXCL5*	2.40		0.211372																
*CXCL9*	7.01		**0.033082**																
*IL15*	2.91		0.075476																
*CXCL8*	5.84		0.184166																
*NAMPT*	2.70		0.109591																
*VEGFA*	2.21		0.273501																
*ACTB*	3.32		0.933359																
*B2M*	2.63		0.747371																
**Genes under-expressed vs. IL-1β/IFN-γ**																			
**Unstimulated control**								**Extract A Dig.**								**Extract B Dig.**			
**Gene Symbol**		**Fold Reg.**		**p-Value**				**Gene Symbol**	**Fold Reg.**		**p-Value**					**Gene Symbol**		**Fold Reg.**	**p-Value**
*AIMP1*		-5.92		**0.045492**				*CCR6*	-3.33		0.051222					*CXCL5*		-2.45	0.214364
*CCL15*		-3.17		**0.019539**				*CXCL3*	-3.30		0.341900					*CXCL3*		-2.72	0.395621
*IL1A*		-2.23		0.124865				*CXCL9*	-3.97		**0.049707**					*CXCL9*		-7.34	**0.033197**
*IL1R1*		-3.53		0.281305				*ACTB*	-25.04		0.227065					*ACTB*		-8.13	0.243868
																*IL15*		-2.72	0.082732

Fold Reg.: fold Regulation; Extract A Dig. and Extract B Dig.: *Cannabis* extract underwent to simulated gastrointestinal digestion; Bold numbers: p- Values lower than 0.05

**Fig. 4:**
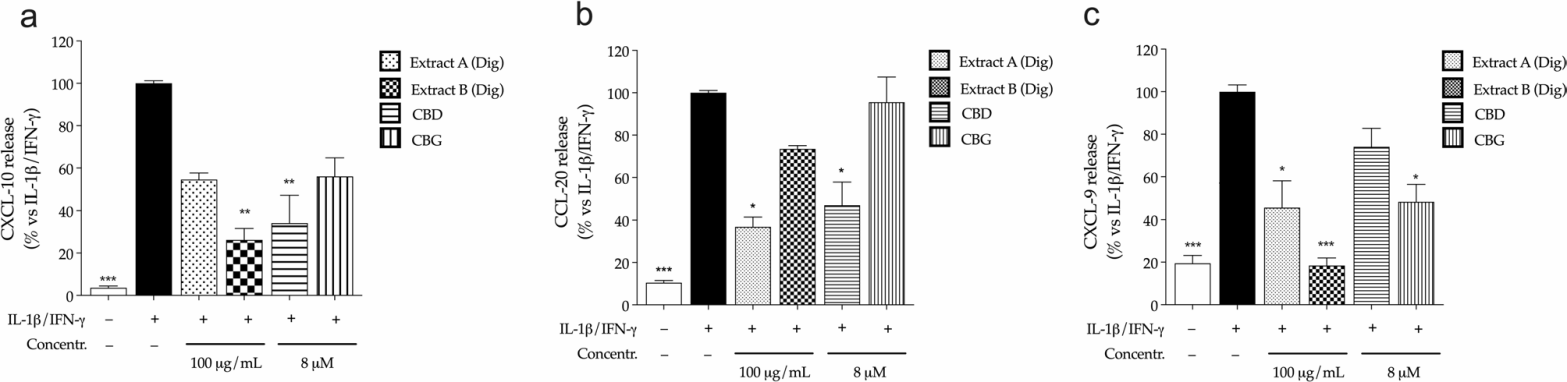
Effect of *Cannabis* extracts underwent to simulated digestion (Extract A Dig., Extract B Dig.) and cannabinoids (CBD, CBG) on the release of CXCL-10 (**a**), CCL-20 (**b**), and CXCL-9 (**c**) in colonocytes (CaCo-2). Chemokine release was measured by ELISA (24 h). Cells were treated with extracts (100 µg/mL) or pure molecules (8 µM) in addition to inflammatory stimuli (IL-1β/IFN-γ), which value was arbitrarily assigned to 100%. Data are expressed as average (%) ± SEM (*n* = 3). *p < 0.05; ** *p* < 0.01; ***p < 0.001 (Kruskal-Wallis test) vs. IL-1β/IFN-γ

Again, in colonocytes, ELISA assays confirmed the significant inhibition of CXCL-10 (Fig. 4a), CCL-20 (Fig. 4b) and CXCL-9 (Fig. 4c) release after the treatment with digested *Cannabis* extracts. The results suggested that the digestion process might improve the bioactivity of Extract B, showing a stronger effect on CXCL-10 and CXCL-9.

For what concerns pure cannabinoids, except for CXCL-10, CBD and CBG showed a different inhibitory pattern, since CBD was more effective against CCL-20, while CBG against CXCL-9. Once again, the overall data suggested that both compounds might contribute, at least in part, to the in the pharmacological activity of the extracts.

Finally, a specific work was conducted to understand the potential role in gut barrier protection. The epithelial barrier was reproduced by Transwell^®^ model and impaired by using the same inflammatory challenge as before (IL-1β and IFN-γ); in addition, a second inflammatory setting based on the co-culture with human macrophages (THP-1) was considered, to involve more complex signals deriving from immune cells.

**Fig. 5:**
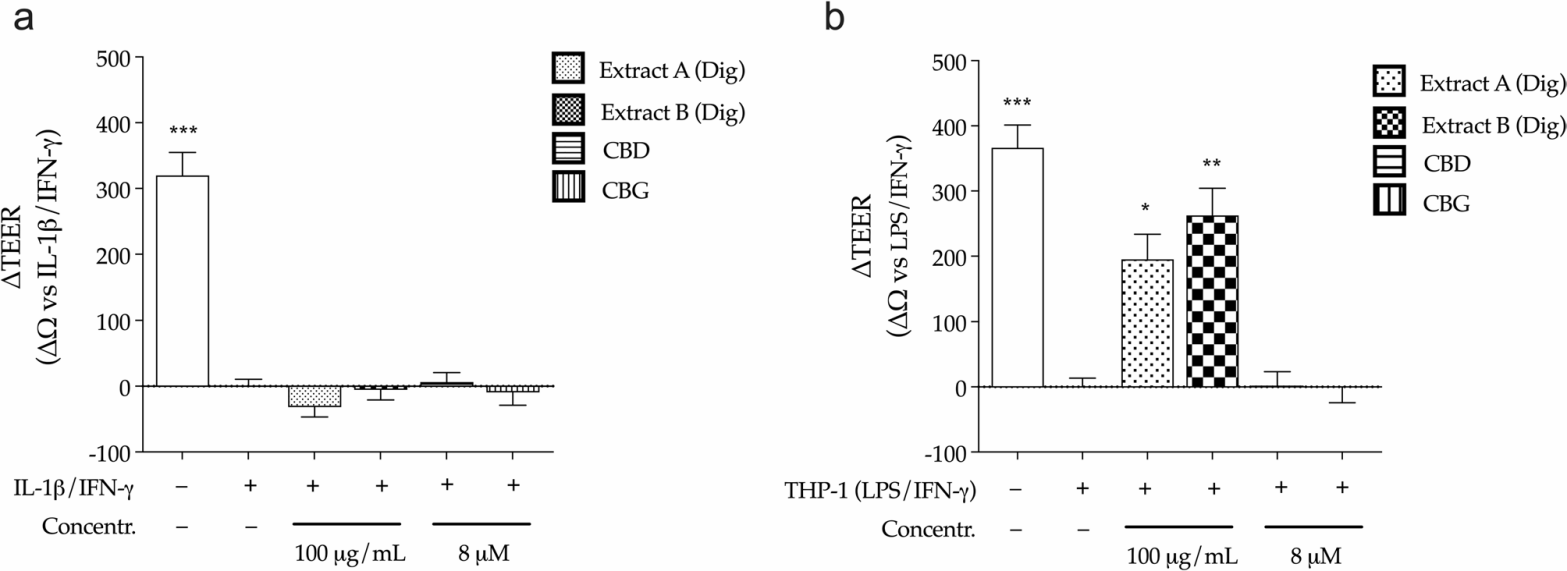
Effect of *Cannabis* extracts underwent to simulated digestion (Extract A Dig., Extract B Dig.) and cannabinoids (CBD, CBG) on epithelial integrity measured by TEER in enterocytes (differentiated CaCo-2). Cells were treated with extracts (100 µg/mL) or pure molecules (8 µM) for 24–48 h, according to two inflammatory settings, respectively: IL-1β/IFN-γ (**a**) and LPS/IFN-γ-induced THP-1 co-culture (**b**). Sodium butyrate 2 mM was used as reference inducer of epithelial integrity (+ 119 Ω and + 219 Ω, respectively). Data are expressed as normalized TEER variation (ΔΩ = Ωt_24/48_-Ωt_0_). Data from independent experiments (*n* = 4) were reported as ΔΩ ± SEM vs. stimulus, to which was arbitrarily attributed the value of 0. *p < 0.05; ** *p* < 0.01; ***p < 0.001 (Kruskal-Wallis test) vs. stimulus

Neither *Cannabis* extracts nor cannabinoids recovered the epithelial integrity, measured by TEER, within the first experimental setting (Fig. 5a). On the contrary, both extracts partially recovered the TEER values within the second experimental setting, thus suggesting an indirect effect involving macrophages (Fig. 5b). Unexpectedly, cannabinoids were not involved in epithelial barrier protection, in contrast to what observed by other authors (Cocetta et al. 2021; Marsh and Smid 2024): this might be related to different experimental settings, including the use of different inflammatory stimuli and lower concentrations of cannabinoids (0.1 and 1 µM, respectively).

**Fig. 6:**
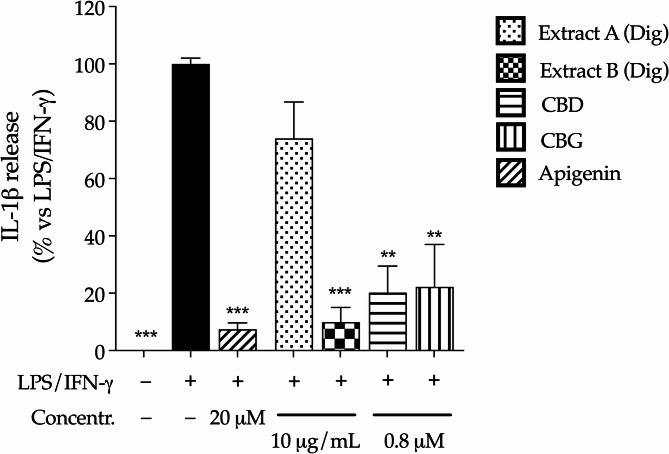
Effect of *Cannabis* extracts underwent to simulated digestion (Extract A Dig., Extract B Dig.) and cannabinoids (CBD, CBG) on the release of IL-1β in macrophages (THP-1). IL-1β release was measured by ELISA (24 h). Cells were treated with extracts (10 µg/mL) or pure molecules (0.8 µM) in addition to inflammatory stimuli (LPS/IFN-γ), which value was arbitrarily assigned to 100%. Apigenin 20 µM was used as reference anti-inflammatory compound (-77%). Data are expressed as average (%) ± SEM (*n* = 3). **p < 0.05, ***p < 0.001 (Kruskal-Wallis test) vs. LPS/IFN-γ

This hypothesis was investigated through additional experiments, in which the single culture of human macrophages was treated with 10 folds lower concentrations of *Cannabis* extracts or pure cannabinoids (10 µg/mL and 0.8 µM, respectively), to reflect the potential availability after intestinal absorption (Mottarlini et al. 2022). In line with previous data collected on colonocytes, extracts and cannabinoids were not cytotoxic in macrophages (Fig. S1c); Extract B inhibited the release of IL-1β more efficiently than Extract A, which exhibited a slight and not significant activity (Fig. 6). Both cannabinoids showed strong inhibitory effect, in line with their well-known suppressive activity on immune cells. This data sustained the hypothesis that the anti-inflammatory properties of cannabinoids only partially explained the peculiar features of *Cannabis* extracts: in fact, their activity on human macrophages was not responsible for the recovery of epithelial barrier, thus remarking the involvement of additional compounds other than CBD and CBG, or complex interactions.

**Fig. 7:**
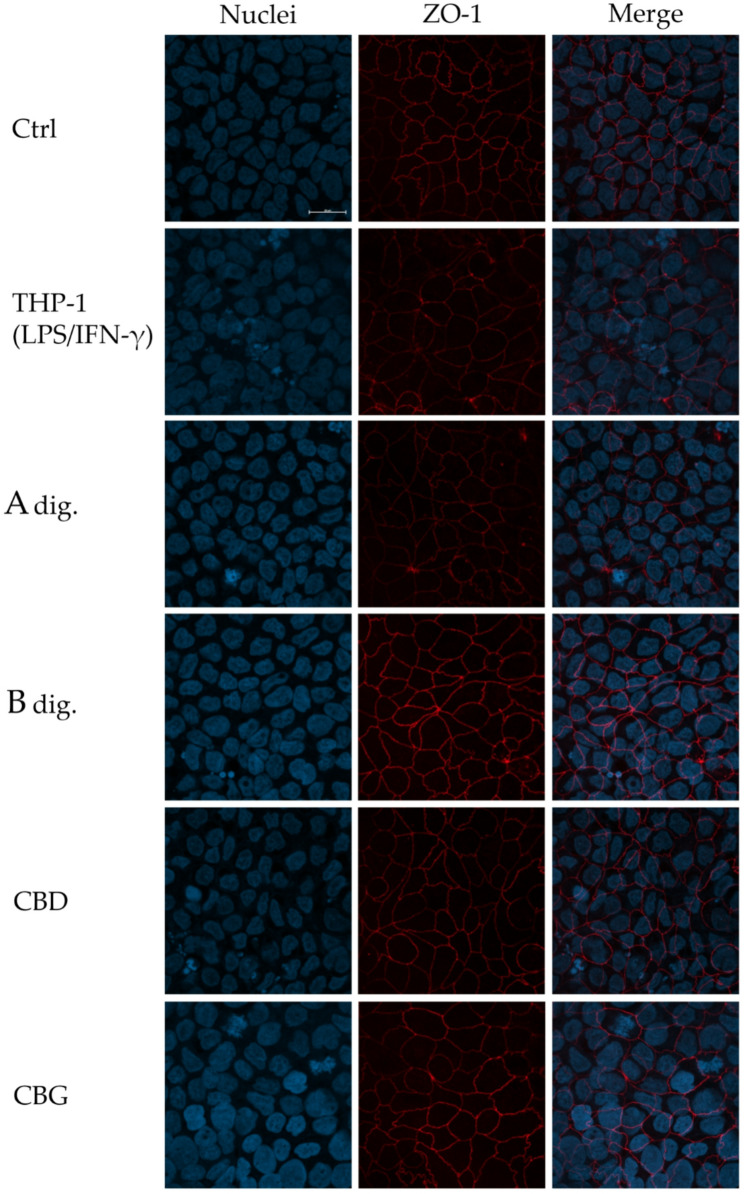
Effect of *Cannabis* extracts underwent to simulated digestion (A Dig., B Dig.) and cannabinoids (CBD, CBG) on the expression and organization of ZO-1, measured by immunofluorescence in enterocytes (differentiated CaCo-2). Cells were co-cultured with human macrophages (THP-1) stimulated with LPS/IFN-γ and treated with extracts (100 µg/mL) or pure molecules (8 µM) for 48 h. Representative images (60x magnification, 20 μm bar scale) report cell nuclei (DAPI, blue color) and membrane-associated ZO-1 (red color), in a separate and merged form

Finally, results were further corroborated by immunofluorescence experiments, evaluating the expression and organization of ZO-1 in the co-culture model, which is dysregulated in inflammatory disorders affecting the intestinal barrier (Vancamelbeke and Vermeire 2017). Again, in line with data showed in Fig. 5b, Extract B prevented the reduction in protein expression and recovered the serrated organization of ZO-1 in comparison to the inflammatory condition (Fig. 7). In analogy, cannabinoids only marginally participated in the biological effect.

## Discussion

Few promising clinical studies indicate that *Cannabis* extracts, containing Δ⁹-THC or CBD might be useful for the control of IBD-related symptoms (Naftali et al. 2013, 2021; Irving et al. 2018). Preclinical studies sustain the plausible role of Δ⁹-THC and CBD as anti-inflammatory and analgesic agents (Pagano et al. 2016; Sun et al. 2024; Cocetta et al. 2021; Schicho and Storr 2012; Jamontt et al. 2010), while minor cannabinoids, such as CBG, have been poorly investigated (Borrelli et al. 2013; Anderson et al. 2024). Of note, the combination of Δ⁹-THC and CBD may exert additional effects in comparison to separate compounds (Jamontt et al. 2010); moreover, synergistic anti-inflammatory effects were also purposed for complex combinations of other natural compounds, typically occurring in *Cannabis* extracts (Sangiovanni et al. 2019; Martinelli et al. 2022; Anil, Peeri, and Koltai 2022).

Although the efficacy and safety of these *Cannabis*-based products still require further investigation (Hasenoehrl, Storr, and Schicho 2017), the relative early age of onset suggests that the use of standardized extracts devoid of Δ⁹-THC might theoretically represent a safer choice for IBD treatment.

Here, we report that two *Cannabis* extracts containing CBD and CBG show a better anti-inflammatory profile and lower cytotoxicity in respect to pure cannabinoids. Indeed, individual compounds (8 µM) or their combination caused a significant reduction of human colonocytes viability (CaCo-2 cells), which was not observed for *Cannabis* extracts (Fig. A1).

These extracts also impair the expression and the release of pro-inflammatory chemokines emerging from a wide PCR array analysis, with robust evidence for CXCL-9 (Table 3; Fig. 4). Since the impaired chemokines are involved in lymphocytes recruitment, the role of *Cannabis* extracts in autoimmune intestinal diseases, including IBD, might be further explored. Moreover, *Cannabis* extracts also counteract the inflammatory damage to intestinal epithelial barrier, with Extract B playing a major role (Figs. 5b and 6). The anti-inflammatory properties are only partially explained by the presence of CBD and CBG, which exert a fundamental role in chemokines reduction, plausibly through the impairment of NF-κB (Fig. 1b), while they are unable to protect the epithelial barrier. Both extracts contained very low amounts of cannabinoids other than CBD and CBG, such as CBC (close to 0.4%), and terpenes (close to 0.1%, or lower, if considered individually) (Tab. S1). Thus, *Cannabis* extracts may have acted through additional mechanisms in respect to pure compounds.

Limitations of our study might have included the use of cancer cells treated with serum-free media, since cannabinoids may exert antiproliferative effect with a variable concentration depending on the presence of serum (Carkaci-Salli et al. 2024). However, serum deprivation aimed to exclude the influence of proliferation on the release of inflammatory markers; for this reason, we considered these limitations as intrinsic biases of our in vitro study.

The two extracts, A and B, come from the mix of the same *Cannabis* varieties (Chemotype III and Chemotype IV), and are standardized In CBD and CBG at the same level (both 1:1 at 5% w/w). The only difference between them is represented by the primary extraction solvent (while the following decarboxylation, solvent removal and filtration are done in the same way). This study shows how the difference in the primary extraction method can impact the biological activity, even when CBD and CBG are standardized at the same level, showing again the importance of the extraction method for the determination of the phytocomplex and the related biological activity.

## Conclusions

At the best of our knowledge, this is one of the few works in which the biological properties of standardized *Cannabis* extracts were compared with their major cannabinoids. More generally, the role of CBG in intestinal inflammation is matter of interest for its non-psychotropics nature, but it was investigated by few articles before. However, our data suggest that the use of *Cannabis* extracts against intestinal inflammation might be preferred in respect to single cannabinoids. Nevertheless, specific studies should be conducted with the aim to translate the evidence to in vivo models.

## Supplementary Information

Below is the link to the electronic supplementary material.

Supplementary Material 1

## Data Availability

All data were included in the manuscript. Raw datasets used and/or analysed during the current study are available from the corresponding author on reasonable request.

## Data availability

The datasets used and/or analysed during the current study are available from the corresponding author on reasonable request.

## Abbreviations

CBD: Cannabidiol
CBG: Cannabigerol
CCL: CC class chemokine
CXCL: CXC class chemokine
DAPI: 4’,6-diamidino-2-phenylindole dihydrochloride
IBD: Inflammatory bowel diseases
IFN: Interferon
IL: Inteleukin
LPS: Lipopolysaccharide
MCT: Medium chain triglyceride
MTT: 3-(4,5-dimetiltiazol-2-il)-2,5-difeniltetrazolio
NF-κB: Nuclear factor kappa-light-chain-enhancer of activated B cells
TEER: Transepithelial electrical resistance
THC: Tetrahydrocannabinol
ZO-1: Zonulin-1
