# Supplementary material for: Efficacy of non-psychotropic Cannabis sativa L. standardized extracts in a model of intestinal inflammation
Source: J Cannabis Res. 2025 Oct 6;7:74. doi: 10.1186/s42238-025-00335-2 (PMC12502145; doi:10.1186/s42238-025-00335-2)
Supplement: Supplementary file 1 — Supplementary Material 1 [file 42238_2025_335_MOESM1_ESM.docx]

**Supplementary**


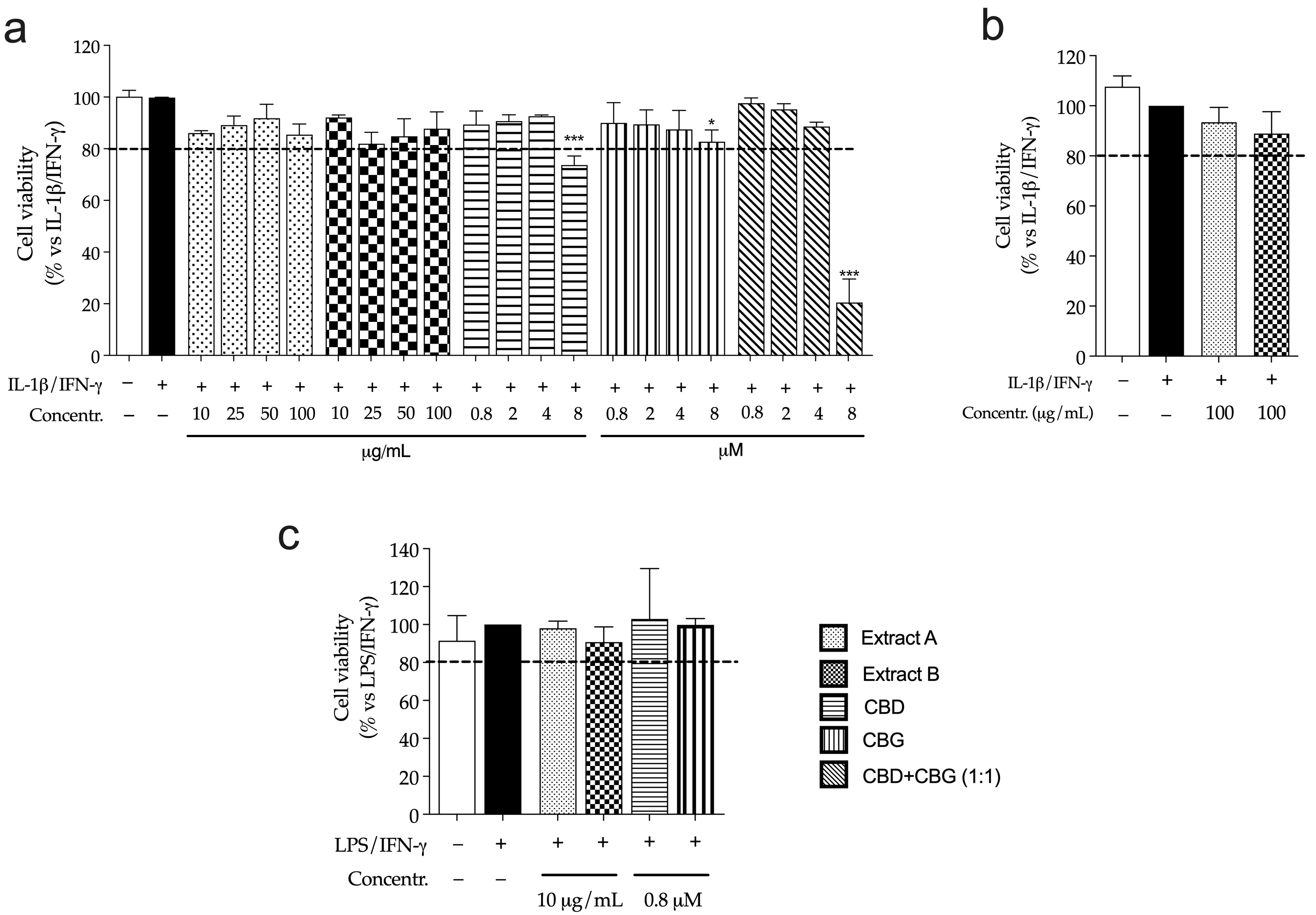


**Figure S1.** Effect of *Cannabis* extracts (Extract A, Extract B) and pure cannabinoids (CBD, CBG, CBD+CBG) on CaCo-2 (**a**, **b**) or THP-1 (**c**) cells viability measured by MTT assay. Extracts were evaluated before (**a**) or after (**b**, **c**) a simulated digestion. Cells were treated for 24 h with extracts or pure molecules in addition to inflammatory stimuli (IL-1β/IFN-γ), which value was arbitrarily assigned to 100 %. Data are expressed as average (%) ± SEM (n=3). Values of viability above 80 % were considered acceptable. *p<0.05, ***p<0.001 (Kruskal-Wallis test) vs IL-1β/IFN-γ.


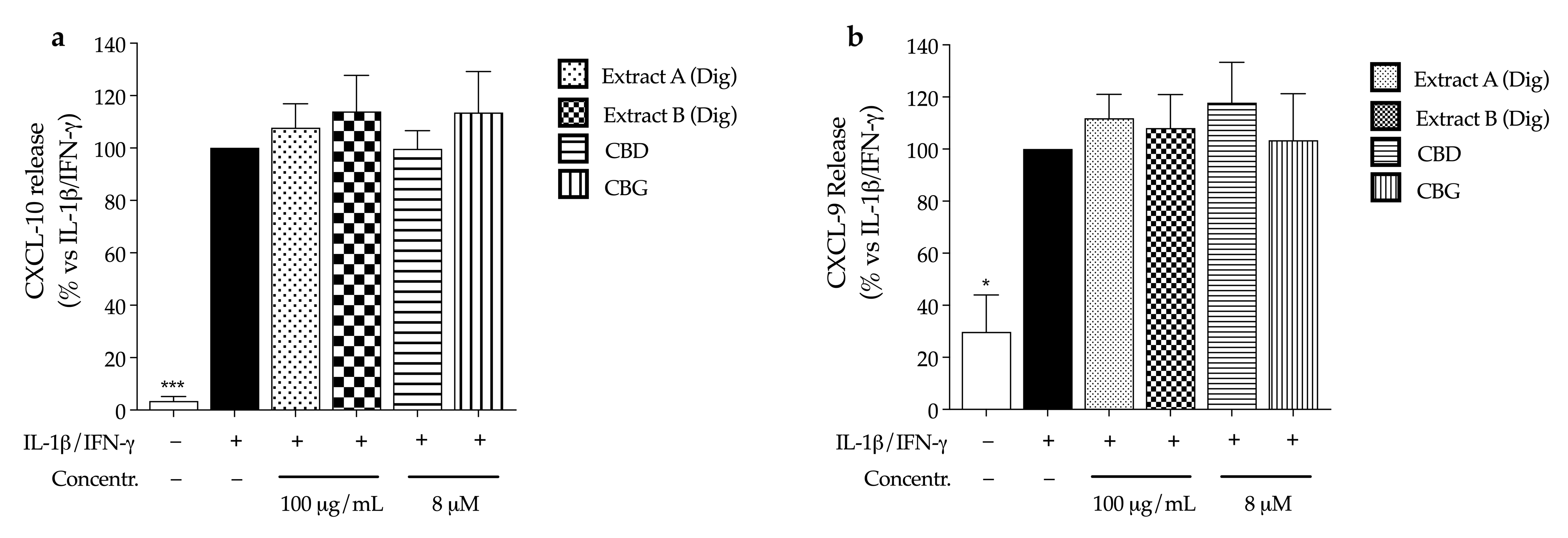


**Figure S2.** Effect of *Cannabis* extracts underwent to simulated digestion (A Dig., B Dig.) and cannabinoids (CBD, CBG) on the release of CXCL-10 (**a**), and CXCL-9 (**b**) in enterocytes (CaCo-2). Chemokine release was measured by ELISA (24 h). Cells were treated with extracts (100 μg/mL) or pure molecules (8 μM) in addition to inflammatory stimuli (IL-1β/IFN-γ), which value was arbitrarily assigned to 100%. Data are expressed as average (%) ± SEM (n=3). *p<0,05; ** p < 0,01; ***p<0,001 (Kruskal-Wallis test) vs IL-1β/IFN-γ.


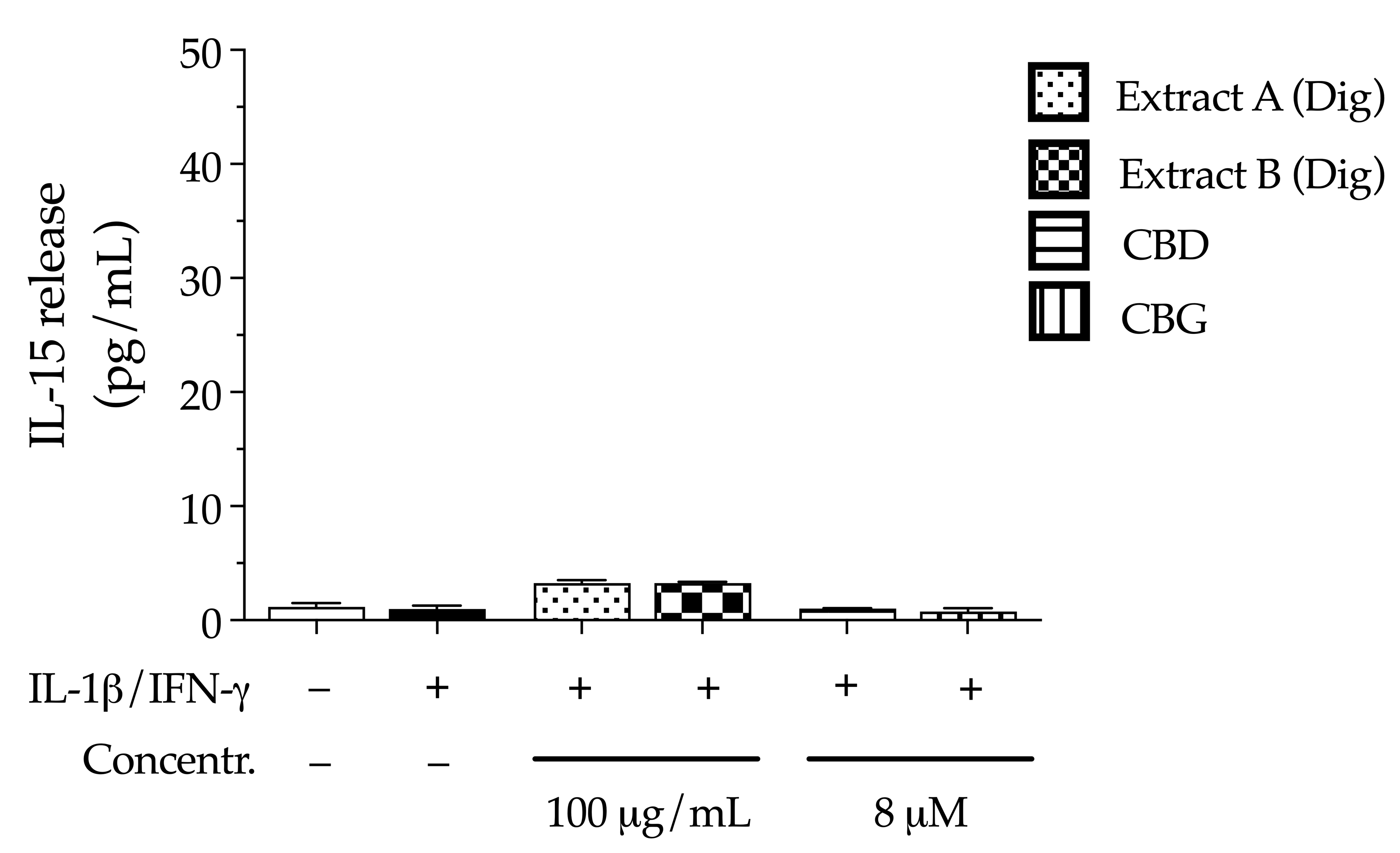


**Figure S3.** Effect of *Cannabis* extracts underwent to simulated digestion (A Dig., B Dig.) and cannabinoids (CBD, CBG) on the release of IL-15 in colonocytes (CaCo-2). IL-15 release was measured by ELISA (24 h). Cells were treated with extracts (100 μg/mL) or pure molecules (8 μM) in addition to inflammatory stimuli (IL-1β/IFN-γ). Data are expressed as average (pg/mL) ± SEM (n=3).

**Table S1.** HPLC-UV and GC/FID analysis of terpenes and cannabinoids from *Cannabis* extracts.

| **Cannabinoids** |  |  | **Cannabinoids** |  |
| --- | --- | --- | --- | --- |
| ***Cannabis* extract A** |  |  | ***Cannabis* extract B** |  |
| HPLC assay **CBD** eq. | 5.19% |  | HPLC assay **CBD** eq. | 5.20% |
| HPLC assay **CBG** eq. | 5.13% |  | HPLC assay **CBG** eq. | 5.11% |
| HPLC assay **CBD** as is | 5.15% |  | HPLC assay **CBD** as is | 5.20% |
| HPLC assay **CBG** as is | 4.88% |  | HPLC assay **CBG** as is | 5.11% |
| Related substances: **CBNR** | 0.07% |  | Related substances: **CBNR** | 0.07% |
| Related substances: **CBDA** | 0.05% |  | Related substances: **CBC** | 0.36% |
| Related substances: **CBGA** | 0.29% |  | Related substances: **D9-THC** | 0.16% |
| Related substances: **CBC** | 0.39% |  |  |  |
| Related substances: **D9-THC** | 0.21% |  |  |  |
| Related substances: **D8-THC** | 0.05% |  |  |  |

| **Terpenes** |  |  | **Terpenes** |  |
| --- | --- | --- | --- | --- |
| ***Cannabis* extract A** |  |  | ***Cannabis* extract B** |  |
| α- Terpineol | 0.07% |  | α- Terpineol | -- |
| β-Caryophyllene | 0.10% |  | β-Caryophyllene | 0.01% |
| α-Humulene | 0.03% |  | α-Humulene | 0.01% |
| Farnesene isomer 3 | 0.05% |  | Farnesene isomer 3 | -- |
| Valencene | 0.02% |  | Valencene | -- |
| Farnesene isomer 2 | 0.05% |  | Farnesene isomer 2 | -- |
| Farnesene isomer 1 | 0.12% |  | Farnesene isomer 1 | 0.02% |
| trans-Nerolidol | 0.01% |  | trans-Nerolidol | 0.01% |
| Caryophyllene oxide | 0.04% |  | Caryophyllene oxide | 0.03% |
| α-Bisabolol | 0.12% |  | α-Bisabolol | 0.11% |
| **Total Terpenes** | **0.61%** |  | **Total Terpenes** | **0.19%** |

**Table S2.** Summary of gene expression measured by PCR array in enterocytes (CaCo-2).

| **Genes over-expressed vs IL-1β/IFN-γ** | | | | | | | | | |
| --- | --- | --- | --- | --- | --- | --- | --- | --- | --- |
| **Unstimulated control** | | | **Extract A Dig.** | | | | **Extract B Dig.** | | |
| **Gene Symbol** | **Fold Reg.** |  | **Gene Symbol** | **Fold Reg.** | **p-Value** |  | **Gene Symbol** | **Fold Reg.** | **p-Value** |
| CCL20 | 10.96 |  | AIMP1 | 3.24 | 0.516586 |  | AIMP1 | 7.83 | 0.181927 |
| CCR6 | 6.40 |  | BMP2 | 2.29 | **0.025343** |  | BMP2 | 2.14 | **0.011355** |
| CSF1 | 16.76 |  | CXCL1 | 2.12 | 0.427203 |  | CCL15 | 2.17 | **0.043512** |
| CXCL1 | 3.81 |  | ACTB | 2.52 | 0.286321 |  | CCL16 | 2.42 | **0.015236** |
| CXCL10 | 140.34 |  |  |  |  |  | CCL23 | 2.70 | **0.015861** |
| CXCL11 | 3.89 |  |  |  |  |  | ACTB | 2.09 | 0.413070 |
| CXCL2 | 4.35 |  |  |  |  |  |  |  |  |
| CXCL3 | 4.13 |  |  |  |  |  |  |  |  |
| CXCL9 | 217.51 |  |  |  |  |  |  |  |  |
| IL15 | 4.57 |  |  |  |  |  |  |  |  |
| CXCL8 | 6.32 |  |  |  |  |  |  |  |  |
| LTB | 3.01 |  |  |  |  |  |  |  |  |
| NAMPT | 3.65 |  |  |  |  |  |  |  |  |
| TNFSF10 | 2.08 |  |  |  |  |  |  |  |  |
| B2M | 4.09 |  |  |  |  |  |  |  |  |

| **Genes under-expressed vs IL-1β/IFN-γ** | | | | | | | | |
| --- | --- | --- | --- | --- | --- | --- | --- | --- |
| **Unstimulated control** | | | **Extract A Dig.** | | | **Extract B Dig.** | | |
| **Gene Symbol** | **Fold Reg.** |  | **Gene Symbol** | **Fold Reg.** | **p-Value** | **Gene Symbol** | **Fold Reg.** | **p-Value** |
| AIMP1 | -3.03 |  | / | / | / | IL1B | -2.73 | 0.281310 |
| C5 | -2.95 |  |  |  |  | TNFRSF11B | -2.90 | 0.058164 |
| CCL23 | -2.26 |  |  |  |  | TNFSF4 | -2.45 | 0.490659 |
| IL1A | -2.53 |  |  |  |  | VEGFA | -6.01 | 0.766059 |
| IL1R1 | -2.17 |  |  |  |  |  |  |  |
| TNFSF4 | -2.46 |  |  |  |  |  |  |  |

Fold reg., fold regulation; Extract A Dig. and Extract B Dig., *Cannabis* extract underwent to gastrointestinal simulated digestion
